# Mesothelin regulates growth and apoptosis in pancreatic cancer cells through p53-dependent and -independent signal pathway

**DOI:** 10.1186/1756-9966-31-84

**Published:** 2012-10-03

**Authors:** Chunning Zheng, Wei Jia, Yong Tang, HuiLiang Zhao, Yingsheng Jiang, Shaochuan Sun

**Affiliations:** 1 General surgery, the affiliated Jinan central hospital of Shandong university, No105, Jiefang Road, District Lixia, Jinan, 250013, R.P China; 2 Hepatobiliary and pancreatic surgery, Huaxi Hospital,Sichuan Univerity, No 37 Guoxue Road, Chengdu, SiChuan, 610041, China

**Keywords:** Pancreatic cancer, Proliferation, apoptosis, Mesothelin, P53

## Abstract

Mesothelin, a secreted protein, is overexpressed in some cancers, including pancreatic cancer. Rescent studies have shown that overexpression of mesothelin significantly increased tumor cell proliferation, and downregulation of mesothelin inhibited cell proliferation in pancreatic cancer cells, but its exact function and mechanism remains unclear. The aim of the present study was to evaluate the effects of mesothelin on proliferation and apoptosis in pancreatic cancer cells with different p53 status and to explore its signal pathway. Mesothelin levels were detected by western blot and RT-PCR assay in human pancreatic cancer AsPC-1, HPAC and Capan-2, Capan-1 and MIA PaCa-2 cell lines. Mesothelin was slienced by shRNA in AsPC-1, Capan-2 and Capan-1 cells with rich mesothelin level, and mesothelin was overexpressed in the HPAC and Capan-2 cells with less mesothelin level. We observed that in the AsPC-1 and Capan-1cells with mt-p53, and Capan-2 cells with wt-p53, shRNA mediated sliencing of the mesothelin significantly increased PUMA and Bax expression and caspase-3 activity, and decreased bcl-2 expression, followed by the reduced proliferation and colony forming capability and increased cell apoptosis. When PUMA was slienced by siRNA in the stable mesothelin shRNA transfected cells, proliferative capability was significantly increased, and apoptosis was decreased. However, in the Capan-2 cells with wt-p53, suppression of the mesothelin significantly increased wt-p53 levels. When p53 was blocked by siRNA in the stable mesothelin shRNA transfected Capan-2 cells, PUMA was inhibited, followed by increased proliferative capability and decreased cell apoptosis. In the HPAC and Capan-2 cells with wt-p53 and in the MIA PaCa-2 cells with mt-p53, overexpression of the mesothelin significantly decreased bax levels and increased bcl-2 levels, followed by increased proliferative and colony forming capability. Furthermore, mesothelin-shRNA-transfected cells exhibited a reduced rate of tumor growth under in vivo conditions. However, mesothelin-transfected cells exhibited a increased rate of tumor growth under in vivo conditions. Our data demonstrated that mesothelin promotes proliferation and inhibited apoptosis through p53-dependent pathway in pancreatic cancer cells with wt-p53, and p53-independent pathway in pancreatic cancer cells with mt-p53. Targeting mesothelin by shRNA is the important method for pancreatic cancer therapy.

## Background

Pancreatic cancer remains stubbornly resistant to many key cytotoxic chemotherapeutic agents and novel targeted therapies. Despite intensive efforts, attempts at improving survival in the past 15 years, particularly in advanced disease, have failed. This is true even with the introduction of molecularly targeted agents, chosen on the basis of their action on pathways that were supposedly important in pancreatic cancer development and progression [1]. Clearly, there is a need to understand more about the molecular mechanisms of pancreatic cancer tumorigenesis and to develop effective treatment strategies for pancreatic cancer.

The mesothelin gene encodes a 69-kDa precursor protein that is proteolytically cleaved into an Nterminus secreted form and a C-terminus membrane-bound form, 40-kDa MSLN, which is a glycosylphosphatidylinositol-linked (GPI)-linked glycoprotein [2]. The normal biological function of mesothelin is unknown. In one study, mutant mice that lacked both copies of the mesothelin gene had no detectable phenotype, and both male and female mice produced healthy offspring, suggesting that mesothelin is not involved in normal growth and development [3]. It has recently found mesothelin is highly expressed in many common epithelial cancers. Mesothelin expression by immunohistochemistry is present in approximately 100% of epithelial malignant mesotheliomas and ductal pancreatic adenocarcinomas, 67% to 100% of ovarian cancers and 41% to 53% of lung adenocarcinomas [4-8]. In addition,mesothelin is expressed to varying degrees by other tumors including cervical, head and neck, gastric, and esophageal carcinomas [9]. This differential expression of mesothelin makes it an attractive target for cancer therapy.

A mesothelin-expressing ascitogenic malignant tumour model that demonstrates morphological features of intraperitoneal tumorigenesis has been created [10]. The tumour model (WF-3)also demonstrates relatively high proliferation and migration rates compared with the parental cell line (WF-0). In pancreatic cancer cells, forced expression of mesothelin significantly increased tumor cell proliferation and migration by 90% and 300%, respectively, and increased tumor volume by 4-fold in the nude mice xenograft model when compared with the vector control cell line [11]. Several studies based on animal or cell culture models indicate that mesothelin expression is involved in the Wnt orβ-catenin signaling pathway, whose deregulation plays an important role in carcinogenesis [12-14]. Bharadwaj et al.has shown that mesothelin-activated NF-κB induces elevated IL-6 expression, which acts as a growth factor to support pancreatic cancer cell survival/proliferation through a novel auto/paracrine IL-6/sIL-6R trans-signaling [15]. Furthermore, mesothelin-induced pancreatic cancer cell proliferation also involves alteration of cyclin E via activation of signal transducer and activator of transcription protein-3 [16], in this study,overexpressing mesothelin in MIA PaCa-2 cells with mt-p53 significantly increased cell proliferation and faster cell cycle progression compared with control cells, and silencing mesothelin in BxPC-3 cells with mt-p53 showed slower proliferation and slower entry into the S phase than control cells [16]. Bharadwaj et al.has recently reported compared to low endogenous mesothelin -expressing MIA PaCa-2 and Panc 28 cells, high endogenous mesothelin -expressing Capan-1(mt-p53), BxPC3(mt-p53), PL 45, Hs 766 T, AsPC-1(null-p53), Capan-2(wt-p53), Panc 48 cells were resistant to TNF-α induced growth inhibition regardless of the p53 status [17]. However, biologic functions and molecular mechanisms that contribute to the tumor progression caused by the overexpressed genes remain largely unknown.

Mesothelin has been implicated as a potential ideal target antigen for the control of mesothelin-expressing cancers such as ovarian cancer, mesothelioma and pancreatic adenocarcinoma.In pancreatic cancer,silencing of mesothelin inhibited cell proliferation and migration in pancreatic cancer cells and ablated tumor progression in vivo and vitro [16]. Vaccination with chimeric virus-like particles that contain human mesothelin substantially inhibited tumor progression in C57BL/6 J mice [11]. Otherwise,knockdown of mesothelin sensitized pancreatic cancer cells to radiation and TNF-a-induced apoptosis [17,18]. However,the molecular mechanisms of mesothelin sliencing on proliferation and apoptosis in pancreatic cancer cells is unclear.

The transcription factor p53 plays a key role in the DNA damage response to genotoxic stress by binding directly to the promoters of target genes and altering the rate at which they are transcribed. Once activated,p53 induces or represses various target genes,including proapoptotic Bcl-2 genes,leading to a myriad of cellular outcomes, including apoptosis,growth arrest, cellular senescence, and DNA repair. Thus, p53 integrates cellular stress responses, and loss of p53 function leads to the aberrant proliferation of damaged cells.It has shown the expression levels of both Bcl-2 and Mcl-1 proteins significantly increased in mesothelin-overexpressed WF-0 transfectants. Interestingly, more endogenous mesothelin introduced caused lower expression of the pro-apoptotic protein Bax. These results indicate that endogenous mesothelin not only enhanced the expression of the anti-apoptotic proteins Bcl-2 and Mcl-1, but also reduced the expression of the pro-apoptotic protein Bax [10].In the present study,we study whether mesothelin regulates proliferation and apoptosis in pancreatic cancer cells through p53-bcl-2/bax pathway.

One important p53 effector is PUMA (p53-upregulated modulator of apoptosis) [19]. PUMA is a Bcl-2 homology 3 (BH3)-only Bcl-2 family member and a critical mediator of p53-dependent and -independent apoptosis induced by a wide variety of stimuli, including genotoxic stress, deregulated oncogene expression, toxins, altered redox status, growth factor/cytokine withdrawal and infection. It serves as a proximal signaling molecule whose expression is regulated by transcription factors in response to these stimuli. PUMA transduces death signals primarily to the mitochondria, where it acts indirectly on the Bcl-2 family members Bax and/or Bak by relieving the inhibition imposed by antiapoptotic members. It directly binds and antagonizes all known antiapoptotic Bcl-2 family members to induce mitochondrial dysfunction and caspase activation [20].

It has shown MIA PaCa-2- mesothelin cells showed increased expression of anti-apoptotic Bcl-xL and Mcl-1,deactivated (p-Ser75) BAD, and activated (p-Ser70) Bcl-2,and vice verce [17]. We hypothesis that mesothelin regulates anti-apoptotic effect via PUMA pathway.

In the present study, we investigated the effect of mesothelin overexpression or sliencing on apoptosis and proliferation in pancreatic cancer cells with different p53 status,and disscused the mechanism.

## Materials and methods

### Cell culture and regents

Human pancreatic cancer cell lines AsPC-1(p53-null), HPAC and Capan-2(wt-p53), Capan-1 and MIA PaCa-2(mutant p53)were purchased from the American Type Culture Collection (ATCC, Rockville, MD). The cells were routinely cultured in Dulbecco’s Modified Eagle’s Medium (DMEM). They were all lemented with 10% fetal bovine serum (FBS) in a 37°C incubator in a humidified atmosphere of 5% CO_2_. Antibodies against the p53 (1:200,DO-1), p21(1:200), caspase-3 (1:100) was from Cell Signaling Technology; PUMA-a(1:200), Mesothelin(1:250)and goat anti-rabbit IgG antibody conjugated to horseradish peroxidase was from Santa Cruz Biotechnology.

### Stable mesothelin shRNA transfection

Mesothelin shRNA Plasmid and shRNA encoding non-effective expression plasmid against GFP (Mock shRNA) were purchased from Santa Cruz,Shanghai,China. Mesothelin shRNA (h) is a pool of 3 target-specific 19-25 nt shRNAs designed to knock down gene expression. For shRNA transfection, AsPC-1and Capan-1/2 cells with rich mesothelin mRNA were were carried out in a 6-well plate. When the cells reached 70% confluence, the transfection process began. Briefly, solution A was prepared by diluting 10 μg of Mesothelin shRNA into 200μL serum-free medium, and solution B was prepared by diluting 20μL Lipofectimine 2000 into 200μLserum-free medium. The two solutions were combined for 20 min at room temperature, and then 0.6 mL serum-free medium was added to the tube containing the complex, and subsequently added to the rinsed cells. The medium was replaced with fresh and complete medium 18 h after the start of transfection. Forty-two hours after transfection, it was replaced with the selective G418 (500-600 ug/mL). Once stable transfections were obtained, the cells were maintained in G418 (250-300 ug/mL). The cells were transfected with either the Mock shRNA or Mesothelin shRNA Plasmid.

### Mesothelin plasmid construction and stable transfection

The full-length ORF of human mesothelin (Genbank accession no. NM 005823)was amplified by PCR from the cDNA of an pancreatic cancer tissue using sense: 5’- GCCAATCACCCTGCACATCAGAGTT -3’, antisense: 5’-TTCCCGTTTACTGAGCGCGAGTTCT-3’. Mesothelin cDNA was digested with *Eco*RI/*Xba*I and cloned in the *Eco*RI/*Xba*I site of pcDNA3.1 following the manufacturer's instructions. Briefly, a tube containing 3 μl of the plasmid and 100 μl of competent *Escherichia coli* was placed on ice for 45 min and then immersed in a 42°C water bath for 90 s without agitation. After transfer of 800 μl of LB broth, the tube was shaken at 150 r/min for 1 h at 37°C, followed by spreading 200 μl of the suspension onto each LB plate containing ampicillin and incubation at 37°C for 16 h. After formation of bacterial colonies, the colonies were picked from the plates and incubated with 5 ml of LB medium containing ampicillin for 16 h. For the extraction of plasmid, 1.5 ml of the bacteria suspension (in an Eppendorf tube) was centrifuged at 12000 r.p.m. for 1 min, then treated with Solution I (50 mmol/l glucose, 25 mmol/l Tris–Cl pH 8.0, 10 mmol/EDTA), Solution II (0.2 N NaOH/1% SDS) and Solution III (mixture of 5 mol/l potassium acetate, glacial acetic acid and H_2_O in the ratio of 6:1.15:2.85), respectively, and centrifuged at 12 000 r.p.m for 10 min. The supernatant was treated with phenol:chloroform (1:1) and centrifuged at 12000 r.p.m. for 10 min at 0°C, then placed at −20°C by adding 2 vol of alcohol for 1 h, followed by centrifugation at 12 000 r.p.m. for 10 min, removal of supernatant and drying at room temperature. Then 20 μl of RNase (100 μg/ml) was added to each tube and incubated at 65°C for 30 min. DNA thus obtained was electrophoresed on 1% agarose gel. Recombinant plasmid was purified by QIA prep spin miniprep kit (QIAGEN). HPAC, Capan-2 and MIA PaCa-2 cells were routinely cultured in DMEM media supplemented with 10% heat-inactivated FBS, 100 μg/ml penicillin and 100 μg/ml streptomycin, and incubated at 37°C in a humidified atmosphere containing 5% CO_2_ in air. Gene transfer was performed according to the manufacturer's protocols. Briefly, ∼3×10^5^ cells/well containing 2 ml appropriate complete growth medium were seeded in a 6-well culture plate, and incubated at 37°C in a 5% CO_2_ incubator until the cells were 70–80% confluent. A cover slip was plated in each well before seeding. After the cells were ringed with serum-free and antibiotics-free medium, the cells were transfected separately with pcDNA3.1- mesothelin cDNA μg/lipofectamine 3 μl (experimental group), pcDNA3.1 1 μg/lipofectamine 3 μl (vector control) and only lipofectamine 3 μl (mock control), followed by incubation at 37°C in a 5% CO_2_ incubator for 6 h. Then the medium was replaced by DMEM culture medium containing 20% FBS. After 48 h, two wells in each group were taken out to detect the transient expression of mesothelin by western blot methods, whereas others were continuously cultured for stable expression of mesothelin. G418 (600-800 mg/l) was added to select the resistant clones after 48 h. Six days later, when most of the cells died, the concentration of G418 was decreased to 300-400 mg/l and cells were cultured for another 6 days. The medium was changed every 3 or 4 days, and mixed population of G418 resistant cells were collected ∼2 weeks later for the examination of stable expression of mesothelin by western blot methods and RT–PCR assay.

### Transient p53 siRNA and PUMA-a siRNA transfection

Small interfering RNA (siRNA) (20 μl) against p53 was purchased from Cell Signaling Technology. Small interfering RNA (siRNA) (10 μl) against PUMA was purchased from Santa Cruz Biotechnology. For transient transfection, 3.3 nM p53 siRNA,PUMA siRNA and their mock siRNA was transfected into stable transfected cells for 48 h in 6-well plates using Lipofectamine 2000 Reagent (Invitrogen)according to the manufacturer’s instructions. At 48 h after transfection, the effects of gene silencing were measured via western blot.

### Xenograft tumors and tissue staining

All animal experiments were approved by the Institutional Animal Care and Use Committee at the Shandong University. Subconfluent stable pancreatic cancer cells with mesothelin overexpression or shRNA silencing were harvested by trypsinization, and resuspended in DMEM. 2×10^6^ cells were inoculated into the right flank of 5- to 6-week-old male nude mice as described previously [11]. The subcutaneous tumor model, the tumor size was measured every week day for 28 days with calipers to calculate tumor volumes according to the formula(length×width^2^)/2.

### In vivo immunohistochemical staining for Ki-67 and cleaved caspase-3

Tumor samples were fixed in 10% buffered formalin for 12 h and processed conventionally to prepare paraffin-embedded block. Tumor sections (5 μm thick) were obtained by microtomy and deparaffinized using xylene and rehydrated in a graded series of ethanol and finally in distilled water. Antigen retrieval was done in 10 mmol/L citrate buffer (pH 6.0) in microwave at closer to boiling stage followed by quenching of endogenous peroxidase activity with 3.0% H2O2 in methanol (v/v). Sections were incubated with specific primary antibodies, including mouse monoclonal anti-ki-67 (ki-67; 1:250 dilutions; DAKO), rabbit polyclonal anti-cleaved caspase-3 (Asp175; 1:100 dilutions; Cell Signaling Technology) for 1 h at 37°C and then overnight at 4°C in a humidity chamber. Negative controls were incubated only with universal negative control antibodies (DAKO) under identical conditions. Sections were then incubated with appropriate biotinylated secondary antibody (1:200 dilutions) followed with conjugated horseradish peroxidase streptavidin (DAKO) and 3,3′-diaminobenzidine (Sigma) working solution and counterstained with hematoxylin. ki-67 -positive (brown) cells together with total number of cells at 5 arbitrarily selected fields were counted at ×400 magnification for the quantification of proliferating cells. The proliferation index was determined as number of ki-67-positive cells × 100/total number of cells. Similarly, cleaved caspase-3 staining was quantified as number of positive (brown) cells × 100/total number of cells in 5 random microscopic (×400) fields from each tumor, and data are presented as mean ± SE score of five randomly selected microscopic (×400) fields from each tumor from all samples in each group .

### RT-PCR assay

Total RNA was isolated from cells or frozen tissues in all treatment conditions using TRIzol per standard protocol. Total RNA was treated with DNase I (Invitrogen) to remove contaminating genomic DNA. PCR analysis was done using the onestep reverse transcription–PCR kit (Invitrogen). GAPDH was used as an internal control. The following primers were used: Mesothelin:sense: 5’- AACGGCTACCTGGTCCTAG -3’, antisense: 5’- TTTACTGAGCGCGAGTTCTC -3’. GAPDH: sense: 5’-TGATGGGTGTGAACCACGAG-3’, antisense: 3’-TTGAAGTCGCAGGAGACAACC-5’. The PCR conditions consisted of an initial denaturation at 95°C for 3 min, followed by 30 cycles of amplification (95°C for 15 s, 58°C for 15 s, and 72°C for 20 s) and a final extension step of 4 min at 72°C. PCR products were analyzed on a 1.5% agarose gel.

### Western blotting

Total cellular proteins from frozen –tissues or cells after forty-eight hours ‘s transfection of plasmids and shRNA were isolated and the protein concentration of the sample was determined by BioRad DC Protein Assay (Bio-Rad Laboratories Inc., Hercules, CA). Gel electrophoresis was performed on a 4-20% sodium dodecyl sulfate (SDS)-polyacrylamide gradient gel(Bio-Rad). Proteins were subsequently transferred to PVDF Immobilon-P membrane (Millipore) for 1 h at 100 V. Following this, the blot membrane was incubated for 1 h in blocking buffer {5% milk powder in Tris-buffered saline(TBS)-T buffer [20 mM Tris–HCl pH 7.5, 500 mM NaCl,0.05% (v/v) Tween 20]}. The blot membrane was then incubated with an anti-FLAG horseradish peroxidase-coupled monoclonal antibody (Sigma) in TBS-T buffer (1:5000 dilution) for 1 h at room temperature. The membrane was washed 4× 10 min in TBS-T buffer. anti-GAPDH (Ambion) was as a loading control.

### Determination of cleaved caspase 3 in vitro

Cleaved caspase 3 was determined by fluorogenic substrates according to the manufacturer's instructions. cleaved caspase 3 was measured fluorometrically at 510 nm on a microplate fluorescence reader (1420 Victor Multilabel Counter; Wallac, Rodgau-Jugesheim, Germany).

### MTT assay

Cell lines treated with shRNA or/and cDNA were plated at 2 × 10^3^ cells per well in 96-well plates for six days. Cytotoxicity was determined by 3-(4,5-dimethylthiazol-2-yl)-2,5-diphenyltetrazolium bromide assay (MTT, Trevigen,Inc., Gaithersburg, MD) in accordance with the manufacturer’s instructions. Plates were read using a Vmax microplate spectrophotometer (Molecular Devices, Sunnyvale, CA) at a wavelength of 570 nm corrected to 650 nm and normalized to controls. Each independent experiment was done thrice, with 10 determinations for each condition tested. At identical time points,cells were trypsinized to form a single cell suspension. Intact cells, determined by trypan blue exclusion, were counted using a Neubauer hemocytometer (Hausser Scientific, Horsham, PA). Cell counts were used to confirm MTT results.

### Colony forming assay

Clonogenic survival analysis was performed for each cell line after treatment with shRNA or/and mesothelin cDNA. Briefly, cell lines treated with shRNA or/and mesothelin cDNA were trypsinized to generate a single-cell suspension and 1×10^4^ cells were seeded into 60-mm tissue culture dishes. Dishes were returned to the incubator for 14 days before staining with crystal violet. At the end of incubation, colonies were stained with 0.005% crystal violet for 1 h and photographed. Plates were analyzed using Metamorph,in which 5 × 5 stitched images were counted and multiplied to give colony counts for the whole plate. Data from three to four independent experiments were used to generate the survival curves.

### In vitro apoptosis assay by flow cytometry

Cells were washed, resuspended in 0.5 mL of PBS, and 1 AL/mL YO-PRO-1, and propidium iodide were added. Cells were incubated for 30 min on ice and analyzed by flow cytometry (FACScan, Becton Dickinson,Franklin Lakes, NJ), measuring fluorescence emission at 530 and 575 nm. Cells stained with the green fluorescent dye YO-PRO-1 were counted as apoptotic; necrotic cells stained with propidium iodide. The number of apoptotic cells was divided by the total number of cells (minimum of 10^4^ cells), giving the apoptotic fraction. Data were analyzed using CellQuest software (Becton Dickinson). All observations were reproduced at least thrice in independent experiments.

### In vitro and vivo apoptosis assay by TUNEL staining

To evaluate apoptosis in vitro, a terminal deoxynucleotidyl transferase–mediated deoxyuridine triphosphate nick-end labeling (TUNEL) assay was done in accordance with the manufacturer’s instructions (ApopTag kit; Intergen Company). The invo TUNEL assay was done according to the methods described previously [21]. The stained sections of tumors of each group were reviewed, and the Apoptosis Index, determined by TUNEL staining, was determined by counting at least 1000 cells in 5 randomly selected high-power fields (magnification, ×200).

### Statistical analysis

Statistical analyses were done with Student’s *t*-test using GraphPad Software program (San Diego, CA, USA). Two-tailed *P*<0.05 was considered statistically significant.

## Results

### Expression of mesothelin in human pancreatic cancer cell lines

**Figure 1:**
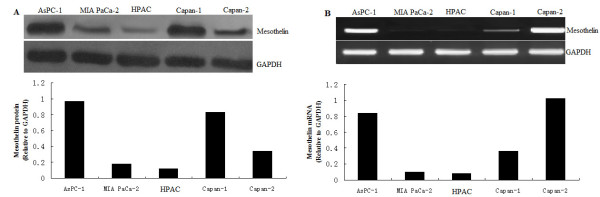
**Expression of mesothelin in pancreatic cancer cell lines.A**. mesothelin protein expression in pancreatic cancer cell lines was detected by Western blot analysis. **B**. Mesothelin mRNA in pancreatic tissues as detected by RT-PCR analysis.

We examined mesothelin expression in AsPC-1(p53-null), HPAC(wt-p53) and Capan-2(wt-p53), Capan-1 and MIA PaCa-2(mutant p53)human pancreatic cancer cell lines by western blot and RT-PCR. In protein levels, rich expression of mesothelin was found in the Capan-1 and AsPC-1 cells, and poor expression was found in the MIA PaCa-2 cells and moderate expression in the Capan-2 cell (Figure 1A). In mRNA level, rich expression of mesothelin was found in the Capan-2 and AsPC-1 cells, and poor expression was found in the HPAC and MIA PaCa-2 cells, and moderate expression in the Capan-1 cell (Figure 1B).

### Generation of mesothelin -expressing or mesothelin sliencing pancreatic cancer cells

**Figure 2:**
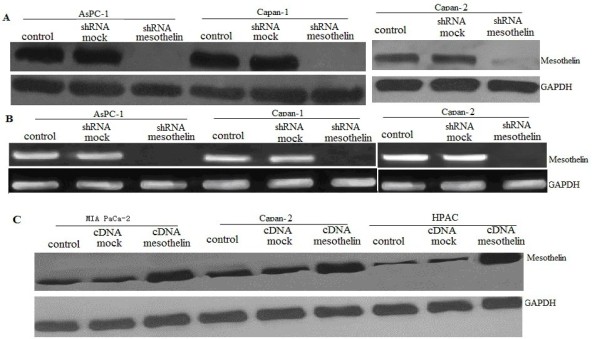
**Mesothelin re-expressing or mesothelin sliencing in pancreatic cancer cells.A**, Whole-cell lysates from mesothelin shRNA-transfected pancreatic cancer cells were subjected to SDS-PAGE and immunoblotted with anti- mesothelin antibody. GAPDH was used as a loading control. **B**, RT-PCR analysis of total RNA (1 μg) isolated from vector control and mesothelin shRNA -transfected pancreatic cancer cells, GAPDH was used as a loading control. **C**, Whole-cell lysates from mesothelin cDNA -transfected pancreatic cancer cells were subjected to SDS-PAGE and immunoblotted with anti- mesothelin antibody. GAPDH was used as a loading control.

AsPC-1,Capan-1 and Capan-2 cells were transfected with mesothelin shRNA or mock shRNA. After 2 weeks of selection with G418, mesothelin -sliencing cells and vector control cells were obtained for each of the two pancreatic cancer cell lines. mesothelin mRNA and protein expression were measured by RT-PCR and Western blot analysis (Figures 2A and B). Mesothelin was knockdown completely in the two cells.

The MIA PaCa-2, HPAC and Capan-2 cells were transfected with pcDNA3.1 mammalian expression vector containing full-length cDNA encoding human mesothelin, or with the empty pcDNA3.1 vector. After 2 weeks of selection with G418, mesothelin-expressing cells and vector control cells were obtained for each of the three pancreatic cancer cell lines. Mesothelin protein expression were measured by Western blot analysis (Figure 2C). All three mesothelin -expressing cells expressed high levels of mesothelin protein, whereas none of the three vector control cell lines expressed detectably increased levels of mesothelin protein (Figure 2C).

### Overexpression of mesothelin increases cell proliferation in pancreatic cancer cells with wt-p53 by p53-dependent pathway

**Figure 3:**
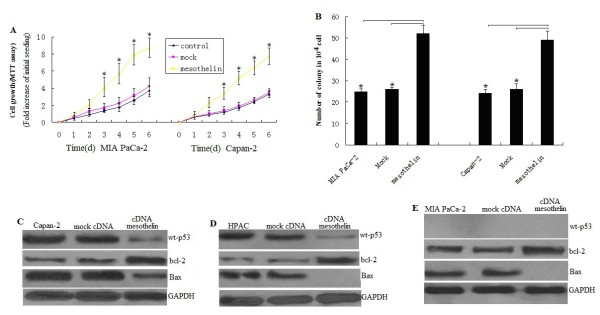
**Overexpression of mesothelin promotes pancreatic cancer cell survival and proliferation.A**, Cell proliferation of MIA PaCa-2 and Capan-2 cells according to MTT assay. Stable mesothelin transfected MIA PaCa-2 and Capan-2 cells and control cells were seeded in 96-well plates (2 × 10^3^ cells/well), serum-starved (0% fetal bovine serum, FBS) for 24 h before changing to 2% FBS growth medium, and cultured for 6 day. Viability was measured with MTT. Relative increase in viability was measured by dividing viability at one time point by viability of the same cell at day 0 (day of addition of growth medium after initial serum starvation) and is plotted along the *Y*-axis. Points, mean of triplicate wells. **B**, cells grown in soft agar were counted. bars, SD. ^*^, *P* < 0.05, relative to control or mock(at 14 days). **C***and***D***, Mesothelin increases* bcl-2 *and decrease Bax* via *p53*-*dependent pathway. Whole cell extract from cells were probed for western blot. ***E***, Mesothelin increases* bcl-2 *and decrease Bax by p53*-*independent pathway. Whole cell extract from cells were detected for western blot*.

To elucidate the role of mesothelin overexpression in pancreatic cancer cell proliferation, we used the 3-(4,5-dimethylthiazol-2-yl)-2,5-diphenyltetrazolium bromide (MTT) assay, comparing the cell growth rate among the mesothelin -overexpressing MIA PaCa-2 stable cell line, the empty vector MIA PaCa-2 stable cell line, and the unrelated MIA PaCa-2 cell line. The MTT assay showed that Mesothelin transfected cells proliferated almost 3.1 times faster than the control cells at day 3 (P < 0.05; Figure 3A), and almost 2.6 times faster at day 6 (P < 0.05; Figure 3A). To confirm the role of mesothelin in cell proliferation, we did the above assay with another stably mesothelin -overexpressing pancreatic cancer cell line, Capan-2. The similarity of the results provides further evidence for the role of mesothelin in inducing cell proliferation (Figure 3B). The similarity of the results was also found in HPAC cells (data not shown).

Colony formation assay shown mesothelin overexpression caused about 50% increase in colony formation in mesothelin -overexpressing MIA PaCa-2 and Capan-2 stable cell line compared to control or mock transfected cells at 14 days’ culture,respectively (Figure 3B, *P*<0.05). The similarity of the results was found in HPAC cells (data not shown). This result further suggests the enhanced cell proliferation ability and survival efficiency of mesothelin overexpressed cells.

We next investigated the signal transduction mechanism of cell survival and proliferation in these cells of mesothelin-overexpression. To identify signals activated by mesothelin, we examined transcription factors p53, bcl-2,bax and PUMA level in stable mesothelin overexpressed cells.In the HPAC (wt-p53) and Capan-2(wt-p53) cells, mesothelin significantly decreased the p53,bax and increased bcl-2 levels (Figures 3C and D). Although PUMA was a little decrease,no significant different was seen(data not shown). This data indicated mesothelin promotes cell survival and proliferation by p53dependent pathway in HPAC and Capan-2 cells with wt-p53.

### Overexpression of mesothelin increases cell proliferation in pancreatic cancer cells with mt-p53 by p53- independent pathway

**Figure 4:**
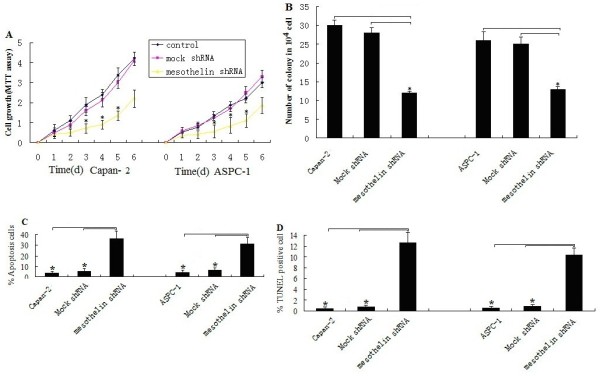
**Mesothelin sliencing suppresses cell survival, proliferation and promotes apoptosis.A**, Cell viability was reduced upon mesothelin sliencing in ASPC-1 and Capan-2 cells. **B**, Number of colony formation was reduced upon mesothelin sliencing in ASPC-1 and Capan-2 cells. **C**, Apoptotic percentages of FCM assays in mesothelin sliencing in ASPC-1 and Capan-2 cells. **D**, Apoptotic percentages of TUNEL assays in mesothelin sliencing in ASPC-1 and Capan-2 cells. Results are means±S.E.M. *P < 0.05.

In the MIA PaCa-2(mutant p53) cells, mesothelin increases bcl-2 levels and decreased bax level,however,the level of p53 and PUMA was not affected (Figure 4E). This data indicated mesothelin promotes cell survival and proliferation by p53-independent pathway in MIA PaCa-2 cells with mt-p53

### Knockdown of mesothelin expression by shRNA inhibited cell growth and induced apoptosis

To determine whether mesothelin could be an effective therapeutic target for pancreatic cancer, the effect of mesothelin shRNA on cell growth of the pancreatic cancer cells was examined in ASPC-1 and CaPan-1/2 pancreatic cancer cells. The reason for choosing these pancreatic cancer cell lines was due to the fact that these cell lines showed much higher expression of mesothelin. The cell viability was determined by MTT, and the effect of mesothelin shRNA on the growth of cancer cells is shown in Figure 4A. We found that down-regulation of mesothelin expression significantly caused cell growth inhibition in the ASPC-1 and CaPan-2 pancreatic cancer cell lines (Figure 4A, *P*<0.05,respectively). Similar results was shown in CaPan-1 cells (data not shown).

Colony formation assay shown mesothelin knockdown of mesothelin caused 50% and 60% decrease in colony formation in mesothelin -sliencing ASPC-1 and Capan-2 stable cell line compared to mock transfected cells,respectively (Figure 4B, *P*<0.05,respectively). This result further suggests the decreased cell proliferation ability and survival efficiency of mesothelin down-expressed cells. Similar results was shown in CaPan-1 cells (data not shown).

To investigate whether the growth-inhibitory effects of mesothelin shRNA are partially related to the induction of apoptosis, the effect of mesothelin shRNA on apoptotic cell death was examined using an FCM and TUNEL assay. These results provided convincing data that down-regulation of mesothelin induces apoptosis in the two pancreatic cancer cell lines (Figures 4C and D). These data suggest that the growth-inhibitory activity of mesothelin down-regulation is partly attributedto an increase in cell death. Similar results was shown in CaPan-1 cells (data not shown).

### Knockdown of mesothelin suppresses cell survival,proliferation and promotes apoptosis by p53-dependent in pancreatic cancer cells with wt-p53

**Figure 5:**
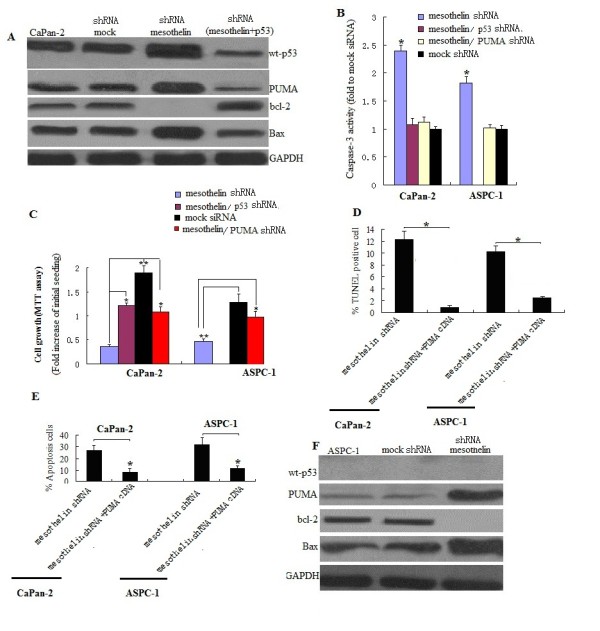
**Mesothelin sliencing suppresses cell survival,proliferation and promotes apoptosis by p53-dependent and -independent pathway in pancreatic cancer cells.A**, Western blot assay for p53, PUMA,bax and bcl-2 in Capan-2 cells with wt-p53. Mesothelin sliencing significantly increased the P53,PUMA and bax levels and decreased bcl-2 level. Knockdown of p53 by shRNA(3 days transfection) decreased the PUMA and bax level and increased the bcl-2 level in stable mesothelin silenced CaPan-2 cells. **B**, Determination of caspase-3 activity. Caspase-3 activity was determined by fluorogenic substrates. Caspase-3 activity was measured fluorometrically at 510 nm on a microplate fluorescence reader. Mesothelin sliencing significantly increased the caspase-3 activity. The activity in mock shRNA transfected cells was defined 1.^*^ denote p < 0.05, compared with mock shRNA controls, *t* test. **C**, Cytotoxicity assay was by MTT. .^*^ denote p < 0.05,^**^p<0.01, compared with mesothelin shRNA groups, *t* test. **D**, Cell apoptosis was determined by FCM assay in samples treated with mesothelin shRNA or mesothelin shRNA plus PUMA shRNA. **E**, Cell apoptosis was determined by TUNEL assay in samples treated with mesothelin shRNA or mesothelin shRNA plus PUMA shRNA.^*^ denote p < 0.05, compared with combined shRNA treatment groups, *t* test. **F**, Western blot assay for p53, PUMA,bax and bcl-2 in ASPC-1 cells with mt-p53. Mesothelin sliencing significantly increased the PUMA and bax levels and decreased the bcl-2 level.

It has shown above mesothelin sliencing suppresses cell survival and proliferation.We next investigated the signal transduction mechanism of cell survival and proliferation in mesothelin-sliencing Capan-1, Capan-2 and ASPC-1 cells with wt- and mt- p53 status. To identify signals activated by mesothelin sliencing, we examined transcription factors p53, PUMA, bax and bcl-2. In the Capan-2 cell with wt-p53 cells, mesothelin sliencing significantly increased the p53, PUMA and bax levels (Figure 5A), caspase-3 activity (Figure 5B) and decreased bcl-2 levels (Figure 5A). When p53 was knockdown by p53 siRNA transfection (3 days after transfection) in stable mesothelin-sliencing cells, PUMA and bax levels (Figure 5B) and caspase-3 activity (Figure 5B) was significantly decreased. But the bcl-2 level was increased (Figure 5B). This data shown mesothelin sliencing decreased PUMA, caspase-3, bax and increased bcl-2 levels was by p53-dependent pathway in Capan-1 cells with wt-p53.

Cell survival and proliferation assay shown p53 or PUMA re-inhibition by siRNA in stable mesothelin sliencing Capan-2 cells promotes cell survival and proliferation (Figure 5C). This data shown mesothelin sliencing inhibited cell survival and proliferation was by p53-dependent pathway in Capan-2 cells with wt-p53. Similar results was shown in HAPC cells (data not shown).

PUMA is a Bcl-2 homology 3 (BH3)-only proapoptotic Bcl-2 family member and mediates p53-dependent and -independent apoptosis.In our study, PUMA is moderate in Capan-2 cells, mesothelin sliencing significantly increased the PUMA levels (Figure 5A) and caspase-3 activity (Figure 5B) followed by rapid and profound apoptosis (Figure 5D), and PUMA re-inhibition by PUMA siRNA transfection in mesothelin sliencing Capan-2 cells lead to decreased apoptosis (Figures 5D and E). This data shown mesothelin sliencing promotes apoptosis was by p53-dependent PUMA pathway in Capan-2 cells with wt-p53. Similar results was shown in HAPC cells (data not shown).

### Knockdown of mesothelin suppresses cell survival,proliferation and promotes apoptosis by p53-independent in pancreatic cancer cells with mt-p53

**Figure 6:**
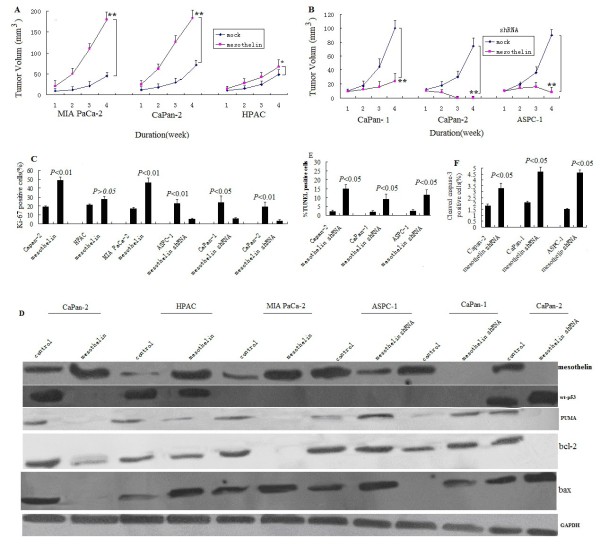
**Effects of mesothelin on pancreatic cancer growth in the xenograft nude mouse model.A**. Subcutaneous tumor volume of HPAC- mesothelin,Capan-2- mesothelin and MIA PaCa-2- mesothelin and their mock cells(2 × 10^6^)were subcutaneously inoculated into nude mice (8 mice per treatment group). Tumor size was measured weekly for 4 weeks. ^**^p < 0.05,^*^p>0.05. **B**. Subcutaneous tumor volume of AsPC-1-shRNA mesothelin, Capan-2-shRNA mesothelin and Capan-1-shRNA mesothelin (2 × 10^6^) were injected into the flank of nude mice (eight per treatment group). Tumor size was measured weekly for 4 weeks. ^**^p < 0.05. **C**, Ki-67-positive cells were counted under ×400 magnifications in five randomly selected areas in each tumor sample. Mean ± SE of 8 tumor samples from individual mouse in each group. **D**, Mesothelin,P53,PUMA,bax and bcl-2 protein was detected by Western blot in tumor samples. **E**, Mesothelin regulates cell apoptosis in orthotopic pancreatic cancer tumors. TUNEL-positive cells were counted under ×400 magnifications in five randomly selected areas in each tumor sample. Mean±SE of 8 tumor samples from individual mouse in each group. **F**, Cleaved capase-3-positive cells were counted under ×400 magnifications in five randomly selected areas in each tumor sample. Mean ± SE of 8 tumor samples from individual mouse in each group.

In ASPC-1 cells with mt-p53, mesothelin sliencing significantly increased PUMA and bax levels (Figure 5F) and caspase-3 activity (Figure 5B), but decreased bcl-2 levels (Figure 5F). PUMA re-inhibition by PUMA siRNA transfection in mesothelin-sliencing ASPC-1 cells lead to increased survival (Figure 6C), decreased apoptosis (Figures 5D and E) and caspase-3 activity (Figure 5B). This data shown mesothelin sliencing promotes apoptosis and inhibits survival was by p53-independent pathway in ASPC-1 cells with mt-p53. Similar results was shown in CaPan-1 cells(data not shown).

### Mesothelin contributes to pancreatic cancer progression in the nude mouse xenograft model

Li et al [11]has reported mesothelin significantly increased tumor cell proliferation in MIA PaCa-2(mutant p53)human pancreatic cancer cell, and mesothelin shRNA significantly decreased tumor cell proliferation in BxPC-3 (mutant p53)human pancreatic cancer cell in vivo and vitro. In the present study, we investigated the effect of Mesothelin sliencing or overexpression on human pancreatic cancer cell lines AsPC-1(p53-null), HPAC and Capan-2(wt-p53), Capan-1 and MIA PaCa-2 (mutant p53) in vivo, and discussed the mechanism. MIA PaCa-2(mt-p53)- mesothelin cells showed a dramatic increase (3.0-fold) in tumor volume over MIA PaCa-2 -mock control cells in the subcutaneous tumor model (p < 0.01,Figure 6A), this was similar to Li’s study [11]. Similarly, CaPan-2- mesothelin (wt-p53) cells significantly increased tumor size by 2.4-fold after 4 weeks compared with mock control cells (p < 0.01, Figure 6A), however, no significant increase was shown in HPAC cells (p > 0.05, Figure 6A).

In contrast, ASPC-1-shRNA mesothelin cells with reduced mesothelin expression showed a significant reduction in tumor volume compared with mock control cells (p < 0.01, Figure 6B). Similarly, CaPan-1- shRNA mesothelin (mt-p53) cells significantly decreased tumor size by 3.4-fold, and CaPan-2- shRNA mesothelin (wt-p53) cells significantly decreased tumor size after 4 weeks compared with mock control cells (p < 0.01, Figure 6B).

Next we examined pancreatic cancer tumors by immunohistochemical methods for the possible antiproliferative, and proapoptotic effects of mesothelin that could have mediated its overall antitumor efficacy. The microscopic examination of ki-67 staining of tumors showed weak ki-67 immunoreactivity in mesothelin shRNA treated ASPC-1, CaPan-1 and Capan-2 groups compared with control group,however, strong staining in ki-67 immunoreactivity in mesothelin treated Capan-2, MIA PaCa-2 groups compared with control group,except for HPAC groups (Figure 6C). In the present study, we observed marked inhibitory effect of mesothelin shRNA on bcl-2,and marked promoting effect of mesothelin on bcl-2 (Figure 6D).

Mesothelin shRNA also showed an increase in PUMA and bax levels (Figure 6D) and TUNEL-positive cells in tumors (Figure 6E), the quantification of which showed a 5, 5.0 and 7-fold (P < 0.05) increase in apoptotic index in ASPC-1, CaPan-1 and Capan-2 cells compared with the control group of tumors (Figure 6D).

Next, tumors were analyzed for cleaved caspase-3 immunostaining (Figure 6F), in which Mesothelin shRNA groups showed 1.8, 2.2 and 3-fold (P < 0.05) increase in cleaved caspase-3-positive cells over that of control group. These results confirmed the apoptotic effect of Mesothelin shRNA in tumors, which could have been mediated by the caspase-3 pathway.

Our results shown in Capan-2 cells with wt-p53, mesothelin regulated PUMA, bax and bcl-2 through wt-p53 dependent pathway. In Capan-1, MIA PaCa-2 and ASPC-1 cells with mt-p53, mesothelin regulated PUMA, bax and bcl-2 through wt-p53 independent pathway (Figure 6D).

## Discussion

Mesothelin is a glycoprotein to be largely restricted to mesothelial cells or to epithelial cells of the trachea, tonsils, fallopian tube, and kidneys [21]. Mesothelin has been reported to be a tumour-associated marker in several types of human cancers, including ovarian carcinomas and adenocarcinomas arising from the pancreatico-biliary tract, endometrium, and lungs [22]. Mesothelin has also been reported to interact with CA125 to mediate cell adhesion [23]. Although the biological functions of mesothelin remain largely unknown, there is evidence that mesothelin has the potential as a new cancer biomarker [10] and as a target molecule for gene therapy [24]. Some investigators have reported that mesothelin can be a new marker for the diagnosis of ovarian carcinoma [25] and as a target in mesothelin-expressing tumours [18], including pancreatic cancer [11]. However,the signal transduction pathways induced by mesothelin resulting in cell survival is unclear.

In the present study, we have shown that mesothelin was overexpressed in the human pancreatic cancer cell lines. Increased mesothelin is associated with increased cell proliferation of pancreatic cancer cells in vitro and contributes to tumor progression in the nude mouse xenograft model. Silencing of mesothelin expression significantly decreased cell proliferation and promoted apoptosis in pancreatic cancer cells in vitro and inhibited tumor growth in vivo. We also shown mesothelin mediated cell survival and apoptosis by p53-dependent and independent conditions.

p53 is a critical regulator of the response to DNA damage and oncogenic stress. Loss of p53 function, through mutation or deletion, is a frequent occurrence in human malignancies. Previous experimental works have converged to indicate that the wt-p53 protein would act as a negative regulator of cell growth [26-28] and a suppressor of transformation and tumonigenesis [29].

In the study reported here, we chose HPAC cells which expressed wt-p53 with less endogenous mesothelin, and Capan-2 cells which expressed wt-p53 with moderate endogenous mesothelin. We found that mesothelin overexpression in HPAC and Capan-2 cells is associated with increased cell proliferation followed by decreased wt-p53. p53 re-inhibition by siRNA in stable mesothelin sliencing Capan-2 and HPAC cells promoted cell survival and proliferation.

It has shown the expression levels of both Bcl-2 and Mcl-1 proteins significantly increased in mesothelin-overexpressed WF-0 transfectants. Interestingly, more endogenous mesothelin introduced caused lower expression of the pro-apoptotic protein Bax. These results indicate that endogenous mesothelin not only enhanced the expression of the anti-apoptotic proteins Bcl-2 and Mcl-1, but also reduced the expression of the pro-apoptotic protein Bax [10]. In the present study,we also observed increased bcl-2 expression and decreased bax expression followed by mesothelin overexpression,and vice verse. Furthermore,the expression of bcl-2/bax was p53-dependent. This data shown mesothelin promoted cell survival and proliferation by p53-dependent pathway in pancreatic cancer cells with wt-p53. However, mesothelin did not affect proliferation in HPAC cells in vivo, which suggests that the tumor microenvironment may play an important role.

In MIA PaCa-2 cells with mutant p53 which expressed less endogenous mesothelin,we found that mesothelin overexpression is also associated with increased cell proliferation followed by decreased bax and increased bcl-2. In contrast, in AsPC-1 cells with p53-null and Capan-1 cells with mt-p53 that expressed more endogenous mesothelin, reduction in expression of mesothelin by shRNA stable silencing resulted in decreased cell proliferation and increased bax and decreased bcl-2. When mesothelin was re-expressed in stable mesothelin sliencing cells, cell proliferation and bax expression was increased and bax was decreased(data not shown). However mesothelin did not affect wt-p53 level. Those results indicate that in pancreatic cancer cells with mt-p53 or null-p53, mesothelin regulates proliferation through p53-independent bcl-2/bax pathway.

p53 functions to regulate several pathways, including cell cycle arrest, DNA repair and apoptosis through transcriptional upregulation of proapoptotic Bcl-2 genes, in particular Puma/Bbc3 [30,31]. Loss of p53 protects cells from p53-dependent apoptotic stimuli due to limited PUMA transcriptional upregulation. The induction of apoptosis is a key tumor suppressor function of p53, particularly in those cells which acquire other oncogenic lesions [32]. p53-dependent Puma upregulation has a central role in this response, inducing apoptosis in the transformed cells [20].

In the present study, silencing endogenous mesothelin by shRNA in Capan-2 (wt-p53) cells increased significant apoptosis followed by increased wt-p53, PUMA and caspase-3 activity. When the p53 or PUMA was blocked by transient p53 siRNA or PUMA siRNA transfection in stable mesothelin shRNA transfected Capan-2 cells,the significant reduction of apoptosis was found. In vivo, mesothelin shRNA also promoted apoptosis, followed by increased p53, PUMA expression and caspase-3 activity. Those results indicate that mesothelin silencing promoted apoptosis through p53-dependent PUMA pathway in cells with wt-p53.

However, in AsPC-1(p53-null) and Capan-1 (mutant p53) cells which expressed rich endogenous mesothelin, silencing mesothelin by shRNA also increased significant apoptosis followed by increased PUMA and caspase-3 activity, and PUMA blocked by transient PUMA siRNA transfection reduced apoptosis,no wt-p53 expression was found. The same result was found in vivo. Those results indicate that mesothelin silencing promoted apoptosis through p53-independent pathway in cells with null/mt-p53.

In addition to p53, a number of other transcription factors are implicated in PUMA induction. The p53 homologue p73 can regulate PUMA expression independent of p53 by binding to the same p53-responsive elements in the PUMA promoter in response to a variety of stimuli [33,34]. On the other hand, PUMA transcription is subject to negative regulation by transcriptional repressors, including Slug [35].In the present study,whether PUMA was regulated by other factors need further investigation.

## Conclusion

The present findings provide evidence of a novel biological function for mesothelin and a mechanism by which mesothelin ptomotes proliferation and inhibited apoptosis through p53-dependent pathway in pancreatic cancer cells with wt-p53, and p53-independent pathway in pancreatic cancer cells with mt-p53 or null-p53. Those results indicate that mesothelin is an important factor in pancreatic cancer growth and a potential target for pancreatic cancer treatment. The significant reduction in pancreatic cancer growth by mesothelin shRNA indicated the importance of shRNA blockage and opened a door for shRNA pancreatic cancer therapy that targets MSLN.

## Competing interests

The authors declare that they have no competing interests.

## Authors’ contributions

ZCN and TY carried out the design of the experiments, performed most of experiments and drafted the manuscript. JW and ZHL participated in establishing the nude models. SSC and JYS participated in the experiments of cell culture and molecular biology. ZCN participated in statistical analysis and interpretation. TY and SSC participated in the design of the experiments. All authors read and approved the final manuscript.
